# Ureteroplasty with buccal mucosa graft without omental wrap: an effective method to treat ureteral strictures

**DOI:** 10.1007/s00345-024-04825-5

**Published:** 2024-03-04

**Authors:** Simon U. Engelmann, Yushan Yang, Christoph Pickl, Maximilian Haas, Christopher Goßler, Sebastian Kälble, Valerie Hartmann, Johannes Breyer, Maximilian Burger, Roman Mayr

**Affiliations:** Department of Urology, St. Josef Medical Center, University of Regensburg, Landshuter Straße 65, 93053 Regensburg, Germany

**Keywords:** Buccal mucosa, Ureter, Transplants, Ureteroplasty, Oral mucosa graft

## Abstract

**Purpose:**

Successful treatment options for ureteral strictures are limited. Surgical options such as ileal interposition and kidney autotransplantation are difficult and associated with morbidity and complications. Techniques such as Boari flap and psoas hitch are limited to distal strictures. Only limited case studies on the success of open buccal mucosa graft (BMG) ureteroplasty exist to this date. The purpose of this study was to evaluate the success of open BMG ureteroplasty without omental wrap.

**Methods:**

In this single-center retrospective study between July 2020 and January 2023, we included 14 consecutive patients with ureteric strictures who were treated with open BMG ureteroplasty without omental wrap. The primary outcome was the success of open BMG ureteroplasty. Further endpoints were complications and hospital readmission. Outcome variables were assessed by clinical examination, kidney sonography, and patient anamnesis.

**Results:**

Out of 14 patients, 13 were stricture and ectasia-free without a double-J stent at a median follow-up of 15 months (success rate 93%). No complications were observed at the donor site, and the complication rate overall was low with 3 out of 14 patients (21%) having mild-to-medium complications.

**Conclusions:**

Open BMG ureteroplasty without omental wrap is a successful and feasible technique for ureteric stricture repair.

**Supplementary Information:**

The online version contains supplementary material available at 10.1007/s00345-024-04825-5.

## Introduction

Treatment options for ureteral strictures are limited. Techniques such as Boari flap and psoas hitch have become standard procedures for distal strictures; however, this is not the case for proximal strictures [1]. Complex upper ureteral strictures, not amenable for ureteroureterostomy or pyeloplasty, present a challenge for urologic surgeons. Possible procedures, such as ileal or appendiceal ureteral replacement and renal autotransplantation, can be complicated and are associated with significant morbidity [1, 2]. In recent years, mucosal grafts for open ureteral reconstruction have become more common, but experiences with the technique and outcome are still limited. The technique of open ureteral reconstruction with buccal mucosa graft (BMG) was first described by Naude in 1999, and only few case series have been published since, none reporting more than ten patients and all using omental wrapping [3–9].

Urological oral mucosa graft (OMG) reconstruction techniques, including both lingual mucosa graft (LMG) and BMG, were initially used for urethroplasty. Since 2016, OMGs are recommended as the first choice for urethral reconstruction with graft tissue in the guidelines of the American Urological Association (AUA) [10]. Due to the feasibility of OMGs, promising outcomes, and low rate of complications, there has been increased interest in OMG for ureteroplasty [11]. However, the European Association of Urology (EAU) guidelines for urological trauma have recently added the specification “oral” graft since the 2022 version, placing it as an equal option to autotransplantation and intestinal interposition [12]. Compared to intestinal interposition, OMG has shown superior results regarding complication and success rates [11]. BMG is an ideal tissue for ureter reconstruction due to its properties including its resistance to infection, compatibility with a wet environment and hairlessness. Additionally, it has a thin lamina propria and thick epithelium layer facilitating the imbibition and inosculation [13]. In the past, omental wrapping has been thought to be important for leakage protection and improvement of neovascularization; however, there is a lack of evidence for its necessity.

In this study, we present the results of 14 consecutive patients who underwent open BMG ureteroplasty for complex ureteral strictures. To the best of our knowledge, this is the largest single-center retrospective study on this surgical technique.

## Patients and methods

### Study design and patient selection

**Fig. 1:**
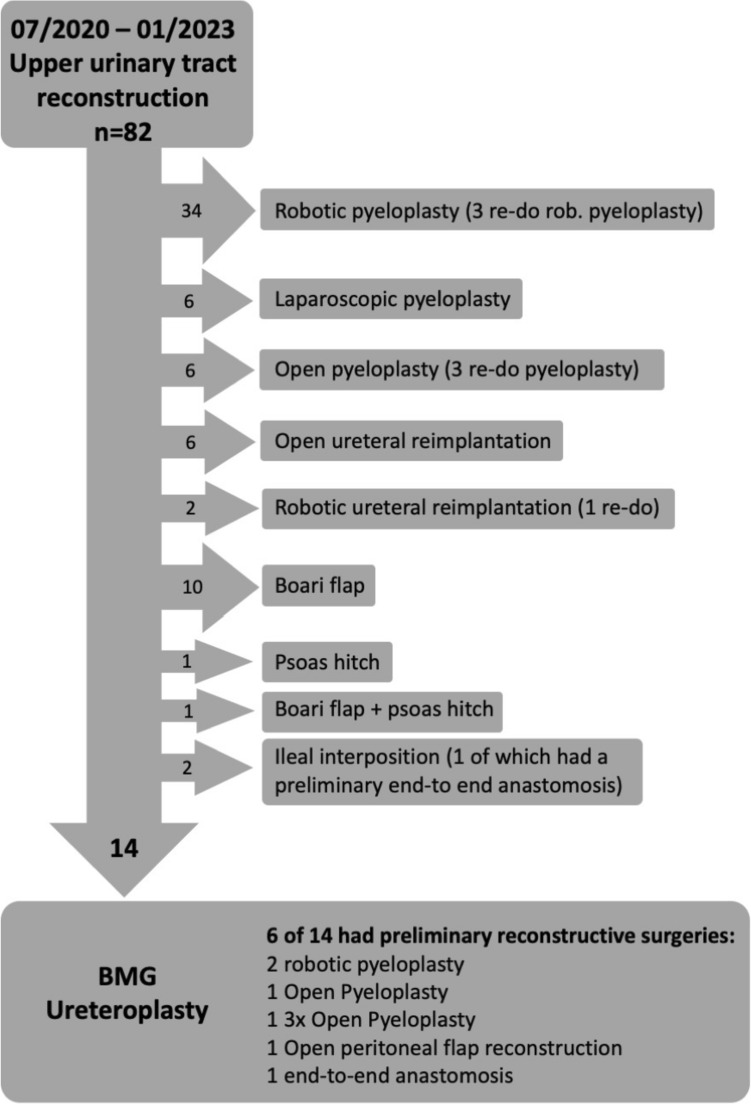
Flowchart of all patients who underwent upper urinary tract reconstruction during the study period

In this single-center retrospective study, 14 consecutive patients were included between July 2020 and January 2023 (Fig. 1). All patients underwent open ureteral reconstruction with BMG onlay graft. The study was approved by the institutional review board of the University Hospital Regensburg. The indication for BMG ureteroplasty was a benign ureteral stricture at the height of the ureteropelvic junction (UPJ), proximal or midureteral, not amenable to orthotopic standard surgical reconstruction due to preliminary reconstructive surgeries, stricture length, and peri-ureteral fibrosis.

### Preoperative preparation

All patients were admitted with or received a urinary drainage in the form of a double-J stent if possible and a nephrostomy in cases where double-J stenting could not be performed due to ureteral occlusion. A preoperative retrograde urography was performed, and in cases with a nephrostomy, antegrade urography was supplemented. Radionuclide renal imaging was performed to evaluate the kidney function of the affected side. All patients underwent urine culture diagnostic preoperatively and were treated perioperatively in case of significant bacteriuria (≥ 100,000 CFU/mL).

### Surgical technique

#### Patient positioning and ureter preparation

**Fig. 2:**
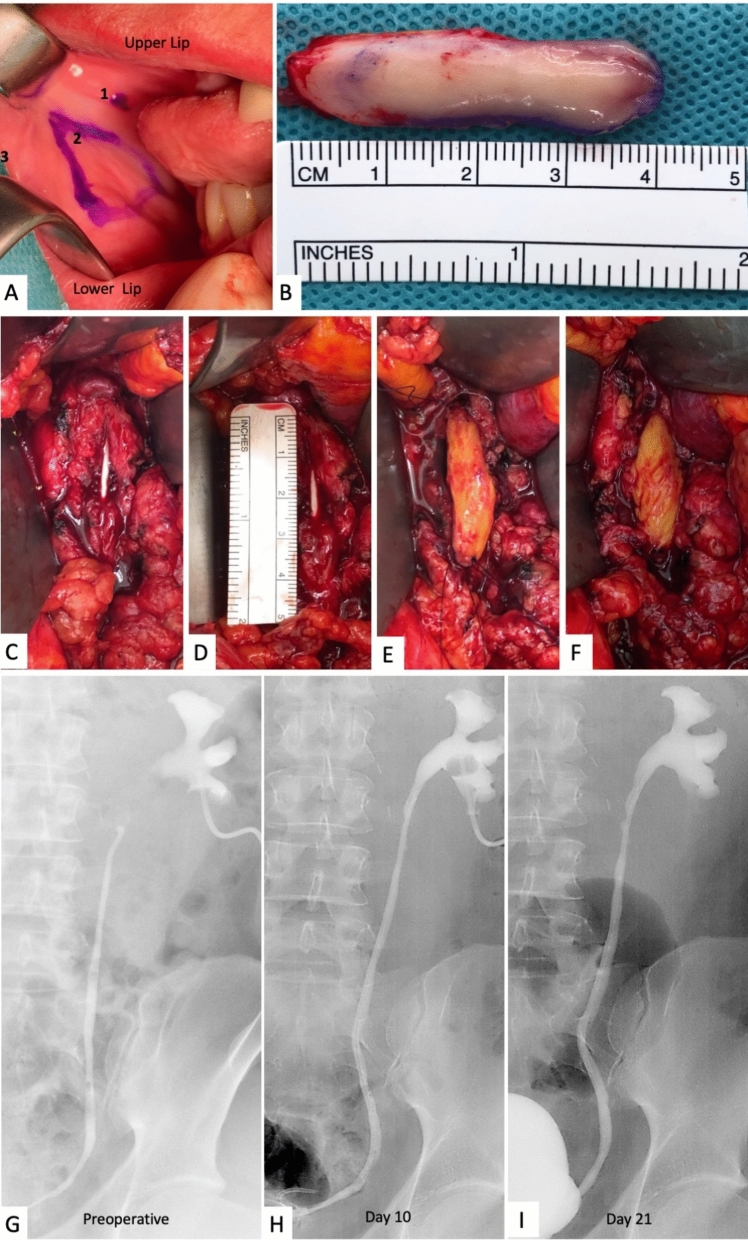
**A**: BMG donor site with “1” parotid duct, “2” buccal mucosa graft (BMG) marked with a pen, and “3” oral commissure. **B**: BMG after harvesting. **C** Lengthwise incised ureteral stricture with double-J stent placed in the ureter. **D** Measurement of defect. **E** onlay buccal mucosa graft (BMG) with sutures placed at the cranial and caudal commissure, and **F** showing the final placement of the BMG with finished continuous sutures along each side. **G**–**I** show a series of X-ray urographies of one patient, preoperative, 10 days postoperative and 21 days postoperative, respectively

All patients received a foley catheter preoperatively. For cases in which the stricture was suspected to involve the UPJ, a flank incision was chosen, and patients were positioned in a lateral flank position on a flexed operation table. All other stricture locations were exposed by a Gibson incision (pararectal incision), and patients were positioned in a supine position with slight elevation on the side of interest. In all cases, surgery was performed strictly extraperitoneal. For better exposition of the operation field, a surgical retractor was placed in situ. The diseased ureter was identified, exposed, and incised on the ventral side along the stricture until reaching the healthy tissue on the proximal and distal side of the ureter over a length of 10 mm on each side. This maneuver was also used in obliterated ureters as shown in the index case (Fig. 2), where a onlay BMG plastic was performed. The exact length of the defect was measured.

#### Harvest of BMG

To facilitate harvesting of the BMG, all patients received general anesthesia by nasotracheal intubation. A mouth retractor and a 3–0 traction suture at the oral commissure were used to expose the inner cheek (Fig. 2). The parotid duct, positioned next to the maxillary second molar, was identified and marked with a surgical sterile pen. Saline solution was injected submucosally, and sharp dissection of the graft borders was performed with a scalpel. When harvesting the BMG, the incision was carefully performed with 1 cm distance from the parotid duct and the vermillion of the oral commissure. The grafts were 1.5 cm in width with varying lengths. Bipolar hemostasis was performed, and the defect was then closed with 3–0 absorbable sutures. A sterile compress saturated with diluted adrenaline was positioned intraorally on the wound surface and was removed before extubation. The graft was prepared ex vivo by removing submucosal tissue.

#### Onlay ureteroplasty and closure

After the incision of the diseased ureter, a 7 French ureteral catheter (double-J stent) was inserted. The anastomosis of the BMG and ureter edges was performed with a 5–0 monofil absorbable running suture (Fig. 2). No omental flap wrapping was performed, but the graft was covered by retroperitoneal or perirenal fat. A drain tube was placed in the operative area, and wound closure was performed accordingly.

### Postoperative management

The drain was removed after 2 days given that its output was < 50 cc/d. The Foley catheter was removed on day 21 after a sufficient retrograde urography by filling the bladder with 200 cc of contrast media (Fig. 2G–I). In patients with a nephrostomy tube, antegrade urography was performed on day 10, and if no extravasation was observed, the nephrostomy tube was removed. The ureteral double-J stent was removed by office cystoscopy 6 weeks after surgery in all cases.

### Follow-up and statistical analysis

Follow-up information was gathered retrospectively using patient records. All patients were followed in our outpatient department. Follow-up included symptomatic assessment of the surgery site and BMG donor site and ultrasound of the urinary tract; the earliest was performed 3 months after removal of the ureteral stent. Follow-up examinations were then continued by local urologists. Success was defined as absence of stricture and ectasia proven by imaging, absence of drainage at follow-up, and absence of symptoms (difficulties whistling, reduced saliva production, flank pain). Descriptive statistical analysis was performed to illustrate patient demographics, perioperative data, and outcomes.

## Results

**Fig. 3:**
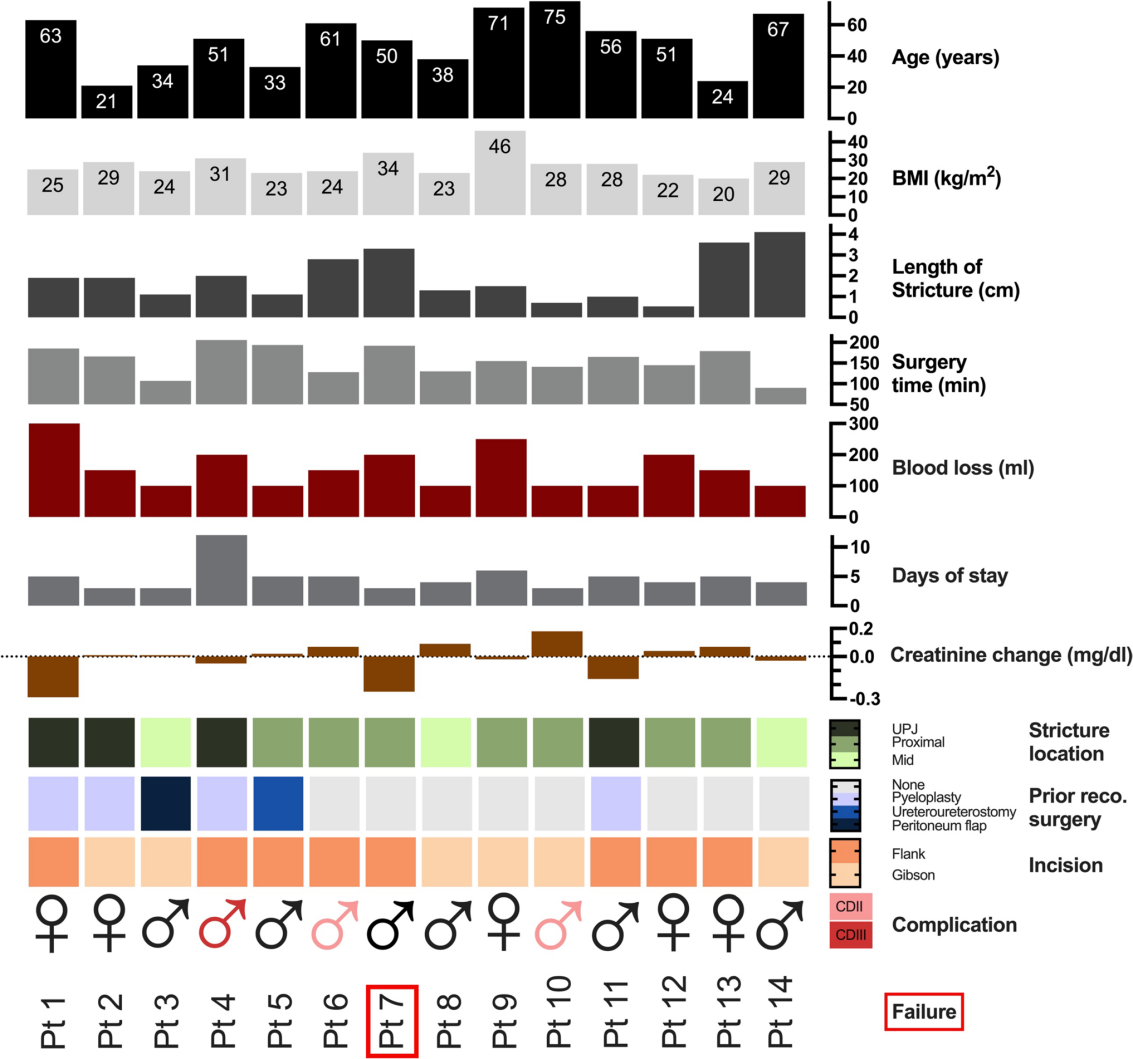
Illustrating the distribution of sex, age, BMI, length of stricture, surgery time, blood loss, days of stay, stricture location, creatinine change, prior reconstructive surgeries, incision used for ureteroplasty with BMG, complications (Clavien–Dindo), and success rate. *Pt* patient, *UPJ* ureteropelvic junction, *reco* reconstructive

Patient characteristics are displayed in Table 1 and Fig. 3. Six of fourteen (43%) patients had a history of failed ureteral reconstruction. Five of these six had their initial surgery performed in other hospitals. The remaining patient had the primary surgery performed in our hospital with a peritoneal flap reconstruction. One patient with congenital UPJO presented with a history of three previous open pyeloplasty surgeries. The median length of stricture was 1.7 cm (range 0.5–4.1). Of 14 patients, four (28.6%) had UPJ strictures, seven (50%) had proximal, and three (21.4%) had midureteral strictures. In most cases (10/14, 71.4%), the etiology of the ureteral stricture was a history of ureteral urolithiasis combined with endoscopic ureteral lithotripsy treatment. All patients apart from one had urinary drainage preoperatively. This one patient rejected double-J stenting due to past experiences, despite having third-degree hydronephrosis.

**Table 1:** Patient characteristics

Characteristic	Outcome
Age, median (IQR)	51 (34–64)
Sex, *n* (%)	
Male	9 (64.3)
Female	5 (35.7)
BMI, median (range)	26.5 (20–46)
Location, *n* (%)	
UPJ	4 (28.6)
Proximal	7 (50)
Middle	3 (21.4)
Laterality, *n* (%)	
Right	8 (57.1)
Left	6 (42.9)
Stricture length (cm), median (range)	1.7 (0.5–4.1)
Etiology, *n* (%)	
Ureteral calculi	10 (71.4)
Iatrogenic during open surgery	2 (14.3)
Congenital UPJO	1 (7.1)
Unknown	1 (7.1)
Preoperative urinary drain	13 (92.9)
Double-J stent	10 (71.4)
Nephrostomy	3 (21.4)
History of endoscopic ureteral lithotripsy, *n* (%)	10 (71.4)
Previous ureteral reconstruction, *n* (%)	6 (42.9)
Pyeloplasty	4 (28.6)
Ureteroureterostomy	1 (7.1)
Peritoneal flap reconstruction	1 (7.1)

*UPJ* ureteropelvic junction, *UPJO* ureteropelvic junction obstruction

The median time of surgery was 160 min (range 90–206). The median length of stay in hospital was 4 days (range 3–12). No operative complications or immediate adverse events occurred. Our standard postoperative pain medication was metamizole 500 mg four times a day for 4 days, one patient received tilidine/naloxon 50 mg/4 mg additionally two times a day for two days, and another patient received Oxycodone 5 mg additionally two times a day for two days. Three patients (21.4%) were readmitted to the hospital, 2 (14.3%) of these were readmitted within 4 weeks after surgery due to urinary infection (Clavien–Dindo II) and treated successfully with antibiotics. One patient was readmitted more than one year after ureteral reconstruction with BMG due to an incisional hernia and was treated accordingly (Clavien–Dindo IIIb).

The overall success rate was 92.9% at a median follow-up of 15 months (range 4–34). One patient had a recurrent stricture 4 months after surgery and was considered a failure. The patient was treated with a double-J stent.

## Discussion

Reconstructive ureteroplasty remains a challenge for urological surgeons. Although open ureteroplasty with BMG was first described in 1999 and since has been proven to be feasible and successful in small study series or case reports, it has not yet become a conventional procedure for the repair of proximal ureteral strictures [3]. Clear recommendations and guidelines are missing. The American Urology Association (AUA) guidelines (2020 amendment) for urotrauma do not even mention OMG (LMG or BMG) [14]. The EAU guidelines recommend OMG as an equal option alongside intestinal replacement and autotransplantation for long proximal strictures [12]. The results of past studies and the current study presenting 14 cases show that ureteroplasty with OMG is a feasible and successful surgical approach, which has little risk of complications and failures. Thus, urological surgeons should consider this procedure for the reconstruction of proximal ureteral strictures, especially in cases where primary reconstructive procedures failed or may not seem achievable.

Intestinal interposition bears many risks including bowel anastomosis leakage, impaired bowel movement or ileus, mucus obstruction of the urinary tract, and recurrent urinary tract infections (UTI) [11, 15]. Additionally, the long-term exposure of urine to ileal mucosa presents a risk for the development of metabolic acidosis [15]. In contrast to this, none of our patients had complications at the BMG donor site, two had a postoperative UTI, and one patient had an incision hernia approximately one year after the procedure. A recent meta-analysis of OMG ureteroplasty versus ileal ureteric replacement by You et al. supports these findings of lower complication rates and less severe complications for OMGs opposed to intestinal interposition [11]. The technique described in this study uses an onlay approach. In cases with complete ureter obliteration, a classical OMG ureteroplasty can be attempted as shown in our index case (Fig. 2). If the obliterated part of the ureter must be resected, an augmented anastomosis with OMG onlay can be necessary. This was performed in a more recent patient and was not included in the study due to the short follow-up period (3 months). Alternatives to OMG ureteroplasty, such as ileal interposition will remain a necessary option in cases where OMG ureteroplasty is not feasible.

Kidney autotransplantation as a therapeutic option for ureteric strictures is a technically complex procedure and, in our opinion, should be reserved as a “last resort” technique before nephrectomy. The complication rates of kidney autotransplantation range from 33 to 46% in the literature [16, 17]. A relatively high rate of transplantation failure (11%) is described in patients undergoing autotransplantation [16]. Other complications often involve the vascular anastomosis (thrombosis, hemorrhages). In contrast to renal autotransplantation, open ureteroplasty with OMG presents a safe and feasible method for ureteral reconstruction. One patient did not achieve long-term recurrent-free reconstruction (patient 7, Fig. 3). Although this patient had no prior reconstructive surgery, he had a relatively long stricture and high BMI, resulting in a difficult surgery, which is reflected in the surgery time of almost three hours. However, none of the patients lost their kidney function, and none had life-threatening complications.

Other alternatives to BMG ureteroplasty, in cases with complicated strictures where orthotopic tissue reconstruction such as re-do pyeloplasty is not available, are appendiceal onlay or tubularized bladder flaps. Drawbacks to appendiceal interposition are the availability of the appendix and major variability of length, with 10–20% insufficiency upon intraoperative assessment [18]. Additionally, a transperitoneal approach is necessary for appendiceal interposition. Opposed to techniques using bladder flaps or bladder transposition, BMG ureteroplasty does not affect the vesicoureteral anti-reflux mechanism.

Up to this date, all published studies on open ureteroplasty with BMG use omental wrap (Supplementary Table A). Omental wrapping is thought to have positive effects on the healing of the anastomosis; it also aids in providing blood supply for the BMG and prevents anastomosis leakage [19]. We could show that perirenal and retroperitoneal fat surrounding the BMG is a sufficient substitution for this mechanism. Not performing omental wrapping allows a strictly extraperitoneal approach, which spares the intestines and reduces the risk of impaired bowel movement or ileus. Despite not performing omental wrapping, we did not observe urinary leakages and had a success rate of 93%. The strictly extraperitoneal approach may even contribute to better neovascularization of the graft considering we did not use omental wrapping.

Recently published studies on OMG ureteroplasty described robotic and laparoscopic approaches and claimed similar success rates to open surgical approaches. In 2022, Liang et al. published a large series on this topic with 41 cases [19]. In all cases, a lingual mucosa graft (LMG) was chosen, 40 patients were operated on a laparoscopic procedure, the success rate was 98%, and the complication rate was low. However, in the described series of patients, the rate of previous failed reconstructive surgery was lower (24%) compared to our current study (43%). Another study by Lee et al. describes rates of 33% prior ureteral reconstruction in a cohort of 54 patients treated with robotic BMG ureteroplasty [20]. The success rate in this study is similar to ours with 87% [21, 22].

Other studies included endpoint radiologic diagnostics such as CT scan, magnetic resonance imaging (MRI), or kidney scintigraphy. Hefermehl et al., for instance, performed a renal scintigraphy 1 year postoperative, showing good renal function at follow-up [9]. We have renounced scintigraphy at follow-up because we believe it is redundant. Restenosis can be detected by renal ultrasound and assessing patient symptoms. Serum creatinine levels give little indication of individual kidney function and may be easily compensated by a healthy unaffected kidney. In our cohort, there was no significant change in creatinine levels preoperatively and at follow-up. We propose the most important parameters of success being symptom-free patients, including symptoms at the donor site, and renal ultrasound.

Our study is not free of limitations. Nowadays, BMG ureteroplasty is performed by the robotic approach in experienced hands, and there is enough literature to support this [22–24]. As the majority of our depicted patients already had open reconstructive surgery before the BMG ureteroplasty, an open approach was attempted to establish the procedure in our department. Future cases will be performed with the robotic platform, to implement our experience on a robotic level. In comparison to other studies (Supplementary Table A), the measured strictures are relatively short, but even a short stricture can be very complex, if an end-to-end anastomosis has been performed and failed. In addition, the method of measuring the stricture is often not stated in published literature and thus not standardized. A further limitation is the follow-up period with a median follow-up of 15 months. Thus, long-term effects such as late graft malignancy cannot be assessed in this study. For ureteroplasty with ileal interposition, the risk of late malignancy has been described in the past, not so, however, for BMG [11, 25, 26].

## Conclusion

To conclude, we presented the largest study on open ureteroplasty with BMG without omental wrapping. This surgical technique is successful and feasible and should be more commonly used as a therapeutic option for ureteric strictures. There is still a great need for standardized guidelines, further promotion, and encouragement to carry out this procedure.

## Supplementary Information

Below is the link to the electronic supplementary material.

Supplementary file1 (DOCX 20 KB)

## Data Availability

The data that support the findings of this study are available from the corresponding author, upon reasonable request.
