# Supplementary material for: Ureteroplasty with buccal mucosa graft without omental wrap: an effective method to treat ureteral strictures
Source: World J Urol. 2024 Mar 4;42(1):116. doi: 10.1007/s00345-024-04825-5 (PMC10912248; doi:10.1007/s00345-024-04825-5)
Supplement: Supplementary file 1 — Supplementary file1 (DOCX 20 KB) [file 345_2024_4825_MOESM1_ESM.docx]

# Supplementary data

## Ureteroplasty with Buccal Mucosa Graft without Omental Wrap: An Effective Method to Treat Ureteral Strictures.

Simon U. Engelmann^1^, Yushan Yang^1^, Christoph Pickl^1^, Maximilian Haas^1^, Christopher Goßler^1^, Sebastian Kälble^1^, Valerie Hartmann^1^, Johannes Breyer^1^, Maximilian Burger^1^, Roman Mayr^1^

^1^Department of Urology, St. Josef Medical Center, University of Regensburg, Landshuter Straße 65, 93053 Regensburg, Germany

Corresponding Author:

Roman Mayr, MD

Department of Urology, St. Josef Medical Center, University of Regensburg, Landshuter Straße 65, 93053 Regensburg

Germany

Email: roman.mayr@ukr.de

**Supplementary table A.** Summary of published literature on open ureteroplasty with BMG (excluding single-case studies) showing surgical approaches and success rates.

| **Literature** | **Stricture length, range (cm)** | **Location of stricture** | **Surgical approach** | **Omental wrap** | **Success *n/n*** |
| --- | --- | --- | --- | --- | --- |
| Naude, 1999[3] | n/A | Mid, prox., UPJ | n/A | yes | 6/6 |
| Shah, 2003[4] | 5-9 | Mid, prox., panureteric | n/A | yes | 5/5 |
| Badawy, 2010[5] | 3.5-5 | Mid, prox. | Extraperitoneal | yes | 5/5 |
| Kroepfl, 2010[6] | 3-11 | Mid, distal | Extraperitoneal transperitoneal | yes | 5/7 |
| Pandey, 2014 [7] | 4-7 | Mid, UPJ | Extraperitoneal | yes | 3/3 |
| Tsaturyan, 2016 [8] | 2.5-5 | Prox., UPJ | Extraperitoneal | yes | 5/5 |
| Hefermehl, 2020 [9] | 3-5 | Prox. | Transperitoneal | yes | 4/4 |
| Present Study, 2023 | 0.5-4.1 | Mid, prox., UPJ | Extraperitoneal | no | 13/14 |
